# Evaluating the efficacy of ceftazidime/avibactam plus amikacin combination therapy as a carbapenem-sparing agent against ceftazidime/avibactam-resistant *Escherichia coli* using a hollow fiber infection model: a proof-of-concept study

**DOI:** 10.1128/aac.01377-25

**Published:** 2026-05-06

**Authors:** Mikaela M. Walker, Jason A. Roberts, Yixuan Li, Saiyuri Naicker, Steven C. Wallis, Jenny L. Ordonez, Hayoung Won, Brian M. Forde, Fekade B. Sime

**Affiliations:** 1 UQ Centre for Clinical Research, Faculty of Health, Medicine and Behavioural Sciences, University of Queensland 1974, Brisbane, Australia; 2 Department of Intensive Care Medicine, Royal Brisbane and Women's Hospital 3883, Brisbane, Australia; 3 Division of Anaesthesiology Critical Care Emergency and Pain Medicine, Nîmes University Hospital, University of Montpellier 27037, Nîmes, France; 4 Institute for Molecular Bioscience, The University of Queensland 1974, Brisbane, Queensland, Australia; 5 School of Pharmacy and Pharmaceutical Sciences, The University of Queensland, Brisbane, Queensland, Australia; Providence Portland Medical Center, Portland, Oregon, USA

**Keywords:** synergy, carbapenem-sparing, avibactam, ceftazidime

## Abstract

Carbapenems, often regarded as the last line of defense in treatment of urinary tract infections and urosepsis caused by *Escherichia coli*, are under pressure from emerging resistance. Thus, it is necessary to investigate carbapenem-sparing agents to mitigate the dissemination of carbapenem resistance. In this study, we aimed to evaluate the efficacy of ceftazidime/avibactam plus amikacin combination therapy against two ceftazidime/avibactam-resistant *E. coli* clinical isolates harboring IMP-4 carbapenemase (CTAP #226 and CTAP #233). To achieve this, we used a hollow fiber infection model (HFIM) with clinically relevant regimens of amikacin (15 mg/kg q24h) and ceftazidime/avibactam (2 g ceftazidime/0.5 g avibactam q8h), which were administered to the HFIM either alone or in combination over a 7-day treatment course. Initial inoculum was ~1 × 10^7^ CFU/mL. Both amikacin and ceftazidime/avibactam monotherapies resulted in bacterial killing (~4 log_10_ and ~3 log_10_, respectively) within the first 8 h; however, regrowth surpassing the baseline was observed over the following 7 days. Conversely, combination therapy resulted in bacterial killing to <the limit of quantification (10^2^ CFU/mL) within the first 48 h, which was sustained for the entirety of the experiment. This study showed that the combination of amikacin and ceftazidime/avibactam was superior to both monotherapies and therefore presents a promising carbapenem-sparing option for the treatment of ceftazidime/avibactam-resistant *E. coli*. Further clinical studies are required to assess the suitability for use *in vivo*.

## INTRODUCTION

*Escherichia coli* is a versatile pathogen with a multifaceted capacity for causing disease. Treatment typically relies on β-lactam antibiotics, though aminoglycosides, trimethoprim/sulfamethoxazole, nitrofurantoin, and fosfomycin may also be utilized (1). Due to the widespread dissemination of antimicrobial resistance, treatment of *E. coli* infections has grown increasingly difficult. In *E. coli* populations that exhibit third-generation cephalosporin resistance, carbapenem antibiotics are often utilized (2). However, widespread use of carbapenem antibiotics, which are considered the last line of defense, contributes to the dissemination of carbapenem resistance (3). Thus, there is an increased need for carbapenem-sparing agents. Ceftazidime/avibactam has been touted as one such option since its approval by the Food and Drug Administration in the United States in 2015 (4–6). Avibactam is a relatively new non-β-lactam β-lactamase inhibitor. It binds to the β-lactamase active site, reducing the availability of binding sites, thereby inhibiting the enzyme activity (5). Avibactam has been found to restore ceftazidime’s efficacy against Ambler class A and class C and some class D β-lactamase-producing bacteria (7). However, it has no effect on Ambler class B β-lactamases, including IMP, NDM, and VIM metallo-β-lactamases (MBLs) (8). Additionally, in *E. coli* specifically, upregulation of efflux pumps, non-functional proteins OmpF and OmpC, and variants of penicillin-binding protein 3 impacts ceftazidime/avibactam resistance (9, 10). To combat this resistance of purposed carbapenem-sparing agents, combination therapies have long been considered.

While there is discourse around the clinical merits of combination therapies, numerous studies have investigated the use of ceftazidime/avibactam in combination with other non β-lactam antibiotics that are commonly used to treat Enterobacterales infections such as the synthetic aminoglycoside, amikacin (11–17). Amikacin binds to the bacterial 30S subunit and induces mistranslation. Of all aminoglycosides, amikacin is purported to be the most widely used as it is resistant to most aminoglycoside modifying enzymes (18). A 2018 systematic review investigated the success of treating urinary tract infection with single-dose amikacin therapy and found clinical cure in 94.5% patients, which was sustained in 73.4% of cases; the vast majority (72%) of all tested isolates were *E. coli* (19). There is some hesitancy with the prescription of high doses and longer courses of amikacin due to the risk of ototoxicity, nephrotoxicity, and rarely, neuromuscular blockade (6). Still, amikacin offers a useful option for treatment of *E. coli* infections, including in empiric combination with β-lactam antibiotics. Numerous studies have investigated the use of both amikacin and ceftazidime/avibactam-based combination therapies for synergy and mutant suppression against Enterobacterales, both *in vitro* and *in vivo*, though rarely against *E. coli* specifically (12, 20, 21). Therefore, in this article, we aimed to evaluate the bacterial killing capacity and resistance suppression capability of ceftazidime/avibactam and amikacin combination therapy as a carbapenem-sparing agent against two clinical *E. coli* strains.

## MATERIALS AND METHODS

### Bacterial isolates, antimicrobial agents, and antibiotic susceptibility testing

Ten clinical isolates of drug-resistant *E. coli* were supplied by Dr. Patrick Harris, University of Queensland Centre for Clinical Research, Brisbane, Australia. From these 10 isolates, 2 isolates were chosen for the hollow fiber infection model (HFIM), CTAP #226 and CTAP #233. These isolates were stored in cation-adjusted Mueller-Hinton broth (CAMHB) with 20% glycerol at −80°C, and fresh isolates were grown on cation-adjusted Mueller-Hinton agar (CAMHA) prior to each experiment.

Prior to the minimum inhibitory concentration (MIC) testing, amikacin (Wako lot WTH2200) stocks were created with sterile Milli-Q water filtered by 0.22 µm polytetrafluoroethylene syringe filter and stored at −80°C. Similarly, prior to the HFIM studies, 1 mL amikacin stock solutions were prepared and stored at −80°C, while ceftazidime/avibactam stock solutions were prepared from ZAVICEFTA 2000/50, lot 2003E2 (Pfizer, Sydney, Australia), as per manufacturer’s instructions and stored at −80°C.

The MIC of ceftazidime/avibactam was performed using Etest (bioMérieux, Lot 1009588580). The MIC of amikacin was performed in replicates of four by broth micro-dilution method in accordance with European Committee on Antimicrobial Susceptibility Testing (EUCAST) (Version 5.0, January 2024). EUCAST clinical breakpoints table (Version 15, dated 01 Jan 2025) was used to define antibiotic susceptibility and resistance. ATCC 25922 strains were used as quality control strains for all antibiotics. The MIC of ceftazidime/avibactam and amikacin was determined following the HFIM using the same method.

### Simulated regimens

The following treatment regimens were simulated in the HFIM experiments: (i) ceftazidime 2 g, avibactam 0.5 g given every 8 h as a 2-h infusion, (ii) amikacin 15 mg/kg given every 24 h as a 0.5-h infusion, and (iii) ceftazidime 2 g, avibactam 0.5 g given every 8 h as a 2 h-infusion with amikacin 15 mg/kg given every 24 h as a 0.5-h infusion.

The pharmacokinetic profiles were developed for patients with bacteremia (22) and sepsis (23). The pharmacokinetic (PK) profile for amikacin was simulated based on the population PK model developed by Romano et al. (23), for patients with sepsis, considering an average body weight of 80 kg, creatinine clearance of 100 mL/min, and a plasma protein binding of 17% (24). The PK profile of ceftazidime/avibactam was simulated using a one-compartment PK model, considering a half-life of 2 h (20, 25) and targeting peak concentration (*C*_max_) of 74 mg/L for ceftazidime at 2 h post-infusion, and *C*_max_ of 14.2 mg/L for avibactam after 2-h infusion of 0.5 g avibactam, as reported for patients with bacteremia by Li et al. (22). According to the product information, the protein binding for ceftazidime was less than 10% and 5.7 to 8.2% for avibactam; therefore, negligible binding was assumed (26).

### Hollow fiber infection model

The HFIM was assembled as previously described (27). In brief, for each dosing regimen, two HFIM circuits were performed per isolate using FibreCell Systems cellulosic cartridges (Catalog C3008, FiberCell Systems, Inc., Frederick, MD, USA) in a 37°C incubator. CAMHB was supplied via peristaltic pumps (Masterflex L/S; Cole-Parmer, Vernon Hills, IL, USA), while drug delivery and continuous circulation between compartments were maintained by Duet Pumps (FiberCell Systems Inc., New Market, MD, USA). Clearances of the drugs were simulated utilizing the flow rates of broth through the hollow-fiber cartridges. In combination arms, a supplementing compartment was utilized to simulate the differential clearance of each drug, as described by Blaser (28).

Prior to each hollow fiber experiment, *E. coli* isolates were cultured overnight on CAMHA at 37°C, then inoculated into CAMHB and adjusted to a density of 10⁸ CFU/mL, corresponding to a 0.5 McFarland standard. The inoculum was subsequently diluted to achieve a final concentration of 10⁷ CFU/mL, which was then introduced into the cartridge. Bacterial samples were collected from the extra-capillary space of the hollow fiber cartridge at 0, 2, 4, 6, 8, 10, 24, 28, 34, 48, 72, 96, 120, 144, and 168 h. Samples were centrifuged at 3,500 *g* for 5 min, and the supernatant was discarded before the samples were resuspended in sterile phosphate-buffered saline to reduce antibiotic carryover. Following appropriate serial dilution, 100 µL of bacterial suspension was plated on drug-free CAMHA to evaluate the bacterial killing capacity. To evaluate resistant subpopulations, samples were also plated on CAMHA containing each drug at concentrations equivalent to two dilutions above the EUCAST MIC breakpoint: ceftazidime/avibactam (32 mg/L), amikacin (32 mg/L), and a combination of ceftazidime/avibactam (32 mg/L) and amikacin (32 mg/L). Colonies from these resistant subpopulations were isolated for DNA extraction and whole genome sequencing. For pharmacokinetic studies, a 1 mL sample was taken over the course of the experiment (0.5, 1, 2, 4, 6, 8, 24, 24.5, 25, 28, 30, 32, 48, 48.5, 49, 52, 54, 56, 72, 72.5, 73, 76, 78, 80, 144, 144.5, 145, 148, 150, 152, and 168 h) and stored at −80°C.

### DNA extraction and whole genome sequencing

DNA was extracted from *E. coli* isolates using DNeasy UltraClean Microbial Kit (QIAGEN). In brief, *E. coli* was grown on CAMHA at 37°C overnight before ¼ 10 µL loop of culture was selected and added to a mix of 300 µL Powerbead Solution and 50 µL SL solution. The solution was vortex mixed for 10 min and centrifuged at 10,000 *g* for 30 s before 270 µL was added to the appropriate well for the QIAcube Connect (QIAGEN). Following extraction, DNA was stored at −80°C.

Extracted DNA samples were sent to Australian Centre for Ecogenomics at the University of Queensland for library preparation and sequencing in accordance with their standard methodology outlined below.

### Library preparation

A commercial DNA preparation kit (Illumina # 20060059) was used for library preparation as per the manufacturer’s procedure, with the exception that the total volume was reduced to accommodate the reaction in a 96-well plate. The workflow was automated using Mantis Liquid Handler (Formulatrix), covering steps from tagmentation through to PCR amplification, followed by the Epmotion (Eppendorf # 5075000301) automated systems for bead clean-up.

Following library preparation, individual libraries were quantified, and quality control (QC) was performed on Agilent TapeStation 4200 (# G2991AA) using the Quant-iT dsDNA HS Assay Kit (Invitrogen) and Agilent D1000 HS tapes (#5067-5582) in accordance with the manufacturer’s protocol.

### Library pooling, QC, and loading

A pooled library was prepared for sequencing from the Illumina DNA Prep libraries combining equal amounts (2 nM per library). The pool concentration was quantified in three replicates using the Qubit dsDNA HS Assay Kit (Invitrogen), and quality control (QC) check was run on the TapeStation 4200 (Agilent #G2991AA) using D1000 HS tapes (#5067-5582) as per the manufacturer’s protocol. Sequencing was performed on the NovaSeq6000 (Illumina) using NovaSeq6000 SP kit v1.5, 2 × 150 bp paired-end chemistry.

### DNA analysis

The raw Illumina sequence read quality was checked using FastQC v0.11.6 (29). Next, sequence reads for each isolate were quality trimmed using Trimmomatic (version 0.36) (30), removing low-quality bases and Illumina adapter sequences. Taxonomic labels assigned using Kraken 2 (31), which determines taxonomy by comparing K-mers within the reads to a reference database. Quality-trimmed sequence read data for each isolate were screened against an NCBI RefSeq database comprised of bacterial, viral, fungal, and eukaryotic genomes to determine species and identify contaminated samples. Quality-trimmed sequence reads for each isolate were assembled using Spades (version 3.14.1) (32) with default parameters. Following assembly, low-quality contigs (contig length < 100 bp; coverage < 20×) were removed. Sequence types (STs) were assigned using mlst (version 2.16.4) (33) and typing schemes available on PubMLST (34). Antimicrobial resistance genes, virulence genes, and stress gene typing were performed *in silico* using AMRFinderPlus v3.10.24 (35). Serotyping was performed using Ectyper v1.0.0 (36), and phylogroups (clermon typing) were performed using clermonTyping (37).

### Measurement of amikacin and ceftazidime/avibactam concentrations

Amikacin and ceftazidime/avibactam in CAMHB were measured by in-house validated ultra-high performance liquid chromatography-tandem mass spectrometry (UHPLC-MS/MS) methods on an Agilent 1290 Infinity II LC system connected to a 6490 Triple Quadrupole Mass Spectrometer (Agilent, Santa Clara, CA, USA). Test samples were assayed in batches alongside calibrators and quality controls (QCs), and results were subject to batch acceptance criteria.

Amikacin calibration standards and QCs were prepared from amikacin sulfate (Sigma Aldrich, St. Louis, MO, USA) in water and drug-free blank CAMHB, respectively. Aqueous calibration standards 25 µL were combined with 25 µL of drug-free blank CAMHB and QCs, and test samples (i.e., 25 µL CAMHB samples) were mixed with 25 µL water to compensate the volume. These were processed by protein precipitation method, adding 50 µL of internal standard working solution (5 mg/L tobramycin in 5% wt/vol trichloroacetic acid). The mixture was vortex-mixed for 5 min and centrifuged for 5 min at 13,200 × *g*. An aliquot of supernatant (3 μL) was injected onto the UHPLC-MS/MS.

The stationary phase for amikacin analysis was Luna Omega Polar C18 (2.1 × 50 mm, 1.6 µm) analytical column (Phenomenex, Torrance, CA, USA) preceded by a C18 UHPLC guard column (Phenomenex, Torrance, CA, USA). Mobile phase A was 0.2% formic acid (vol/vol) in 5 mM ammonium acetate in water, and mobile phase B was 0.2% formic acid (vol/vol) in 5 mM ammonium acetate in 90% acetonitrile. Mobile phases were delivered with a gradient from 2% to 10% of mobile phase B at a flow of 0.2 mL/min followed by column wash and equilibration phase, producing a backpressure of approximately 2,500 psi. Amikacin was detected in positive ionization mode with multiple reaction monitoring (MRM) transitions of *m*/*z* 586.3 → *m*/*z* 163.0 (quantifier) and *m*/*z* 586.3 → 425.2 (qualifier). The internal standard tobramycin was detected with MRM transitions at *m*/*z* 468.3 → 163.0 (quantifier) and *m*/*z* 468.3 → 324.2 (qualifier). The assay range was 0.2 to 100 mg/L, and the assay was linear with 1/[conc.]^2^ weighting for amikacin. The method met validation criteria for intra-batch precision and accuracy and inter-batch precision and accuracy.

Calibration standards and QCs for ceftazidime and avibactam were prepared from ZAVICEFTA 2000/500 (Pfizer Australia, Sydney, Australia) in water and drug-free blank CAMHB matrix, respectively. Aqueous calibration standards 20 µL were mixed with 20 µL of drug-free blank CAMHB, and QCs and test samples (i.e., 20 µL CAMHB samples) were mixed with 20 µL water. These were processed by protein precipitation method with 75 μL of internal standard working solution (10 mg/L of [^2^H_5_]-ceftazidime and 2.5 mg/L of tazobactam in acetonitrile). The mixture was vortexed for 5 min and centrifuged for 5 min at 13,200 × *g*. A volume of 1 μL of the supernatant was injected onto the UHPLC-MS/MS.

The stationary phase for ceftazidime/avibactam was XTerra RP C18 (2.1 × 150 mm, 3.5 µm) analytical column (Waters, Milford, MA, USA) preceded by a SecurityGuard ULTRA C18 guard cartridges (Phenomenex, Torrance, CA, USA). Mobile phase A was 0.1% formic acid (vol/vol) in water, and mobile phase B was 0.1% formic acid (vol/vol) in acetonitrile. Mobile phases were delivered with a gradient from 5% to 95% of mobile phase B over 6.5 min at a flow of 0.3 mL/min, producing a backpressure of approximately 2,500 psi. Ceftazidime was monitored in positive ionization mode at *m*/*z* 547.11→ 467.8 (quantifier) and *m*/*z* 547.11→ 396.0 (qualifier), and avibactam was monitored in negative ionization mode at *m*/*z* 264.03→ 96.0 (quantifier) and *m*/*z* 264.03→ 80.0 (qualifier), respectively. The internal standards [^2^H_5_]-ceftazidime and tazobactam were monitored at *m*/*z* 552.1→ 396.1 (positive ionization mode) and *m*/*z* 229.0→ 138.2 (negative ionization mode), respectively.

The ceftazidime/avibactam assay was quadratic (with 1/[conc.]^2^ weighting) for the concentration range of ceftazidime: 0.5–200 mg/L and avibactam: 0.125–50 mg/L. Inter-batch accuracy and precision of QCs at 1.5/0.375 mg/L, 40/10 mg/L, and 160/40 mg/L were −3.2%, −4.0%, and −2.3% and 3.9%, 3.1%, and 3.0%, respectively, over seven different batches. Incurred sample reanalysis testing met the criteria for both ceftazidime and avibactam (97% and 100% of test samples, respectively, were within 20% of the mean).

Measured concentration-time profiles of study antibiotics from the multiple arms were subsequently fitted to a one-compartment population pharmacokinetic model using Monolix Suite version 2024R1 (Lixoft, Antony, France) to compare the median population predicted profile with the simulated concentration-time profile.

## RESULTS

### Antibiotic susceptibility testing

The baseline ceftazidime/avibactam MIC for isolates CTAP #226 and CTAP #233 was 24 mg/L, while the amikacin MIC for isolates CTAP #226 and CTAP #233 was 8 mg/L.

### Whole genome sequencing

Whole genome sequencing revealed both strains to belong to phylogroup B1 and serotype O8:H19. The isolates were assigned ST 4360, corresponding to the allelic profile adk(9), fumC(86), gyrB(5), icd(1), mdh(9), purA(13), and recA(6), associated with the ST469 complex. Detected virulence factors included the iss gene, espX1, and fdeC. Antimicrobial resistance genes included β-lactamase genes blaTEM-1, blaIMP-4, and blaEC, as well as the AmpC variant blaACT-56 in all strains. Additionally, all strains were found to carry the aminoglycoside acetyltransferase enzymes: aac(3)-lld and aac(6′)-lb4. The baseline and post-treatment genomes for each isolate were genetically identical at the core genome level, with no sequence variation detected (QC in Table S1).

### Hollow fiber infection model

**Fig 1:**
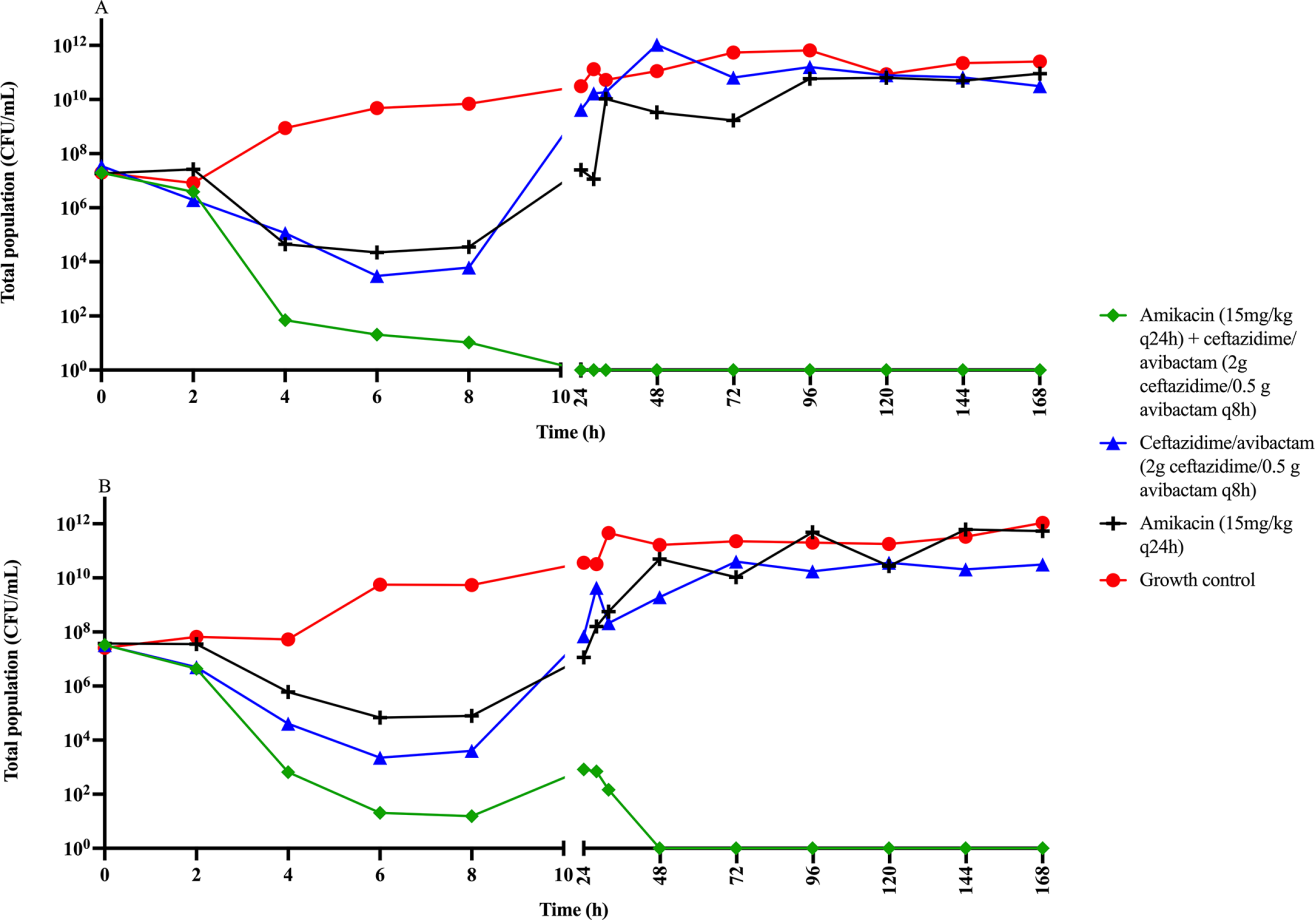
Change in total bacterial population of *Escherichia coli* strains over 168 h in a hollow-fiber infection model with clinically relevant dosing regimens of ceftazidime/avibactam monotherapy, amikacin monotherapy, and ceftazidime/avibactam-amikacin combination therapy. Panels** A **and **B** represent the data for the two study isolates CTAP #226 and CTAP #233, respectively.

The monotherapy of 2 g ceftazidime 0.5 g avibactam exhibited 3 log_10_ bacterial killing within 8 h for both isolates, followed by rapid regrowth to the baseline 10^7^ CFU/mL at 24 h and subsequently to a concentration of ~10^10^ CFU/mL (Fig. 1).

However, the monotherapy of 15 mg/kg amikacin exhibited 4 log_10_ bacterial killing within 8 h, followed by rapid regrowth to 10^7^ CFU/mL (CTAP #233) and 10^9^ CFU/mL (CTAP #226) at 24 h. All strains grew to ~10^10^ CFU/mL when tested with 15 mg/kg amikacin (Fig. 1).

Conversely, all ceftazidime/avibactam and amikacin combination therapies resulted in steady bacterial killing until reaching <LOQ (10^2^ CFU/mL) level at 24 h for isolate CTAP #226 and 48 h for isolate CTAP #233. This was sustained for the entirety of the experiment (Fig. 1).

**Fig 2:**
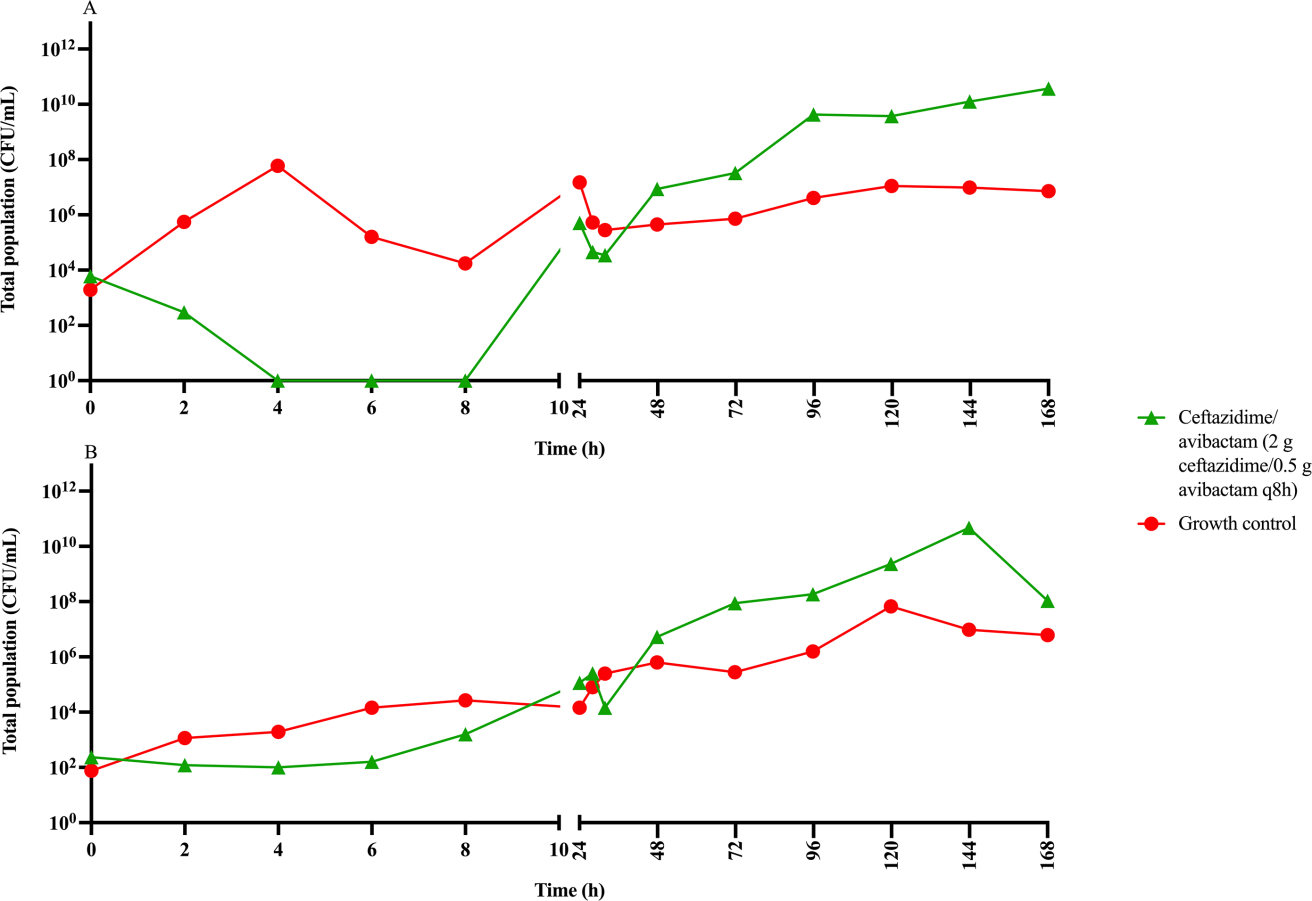
Growth of resistant populations on cation-adjusted Muller-Hinton agar containing 32 mg/L of ceftazidime/avibactam for two clinical strains of *E. coli,* (**A**) CTAP #226 and (**B**) CTAP #233.

Populations resistant to ceftazidime/avibactam were observed from the initial sample taken at 0 h and fluctuated throughout the duration of the experiment when grown on cation-adjusted Muller-Hinton agar containing 32 mg/L of ceftazidime/avibactam (Fig. 2). Neither CTAP #226 or CTAP #233 dosed with 15 mg/kg amikacin exhibited any growth on agar plates containing 32 mg/L amikacin following the HFIM. Similarly, there was no observable growth on plates containing a combination of ceftazidime/avibactam and amikacin.

MIC testing of resistant subpopulations grown on CAMHA containing 32 mg/L of ceftazidime/avibactam (Table 1) revealed more than twofold increase in MIC for duplicates of CTAP #226 and a more than threefold increase in MIC for the duplicates of CTAP #233. There was no growth on any drug plates for isolates dosed with the combination of 2 g ceftazidime, 0.5 g avibactam, and 15 mg/kg amikacin.

**TABLE 1:** Minimum inhibitory concentration of ceftazidime/avibactam (CZA) and amikacin (AMK) for duplicated isolates CTAP #226 and CTAP #233 both before and after the hollow fiber infection model experiment, where available

Strain	Baseline		Post-treatment			
	CZA MIC (mg/L)	AMK MIC (mg/L)	CZA monotherapy	AMK monotherapy	CZA + AMK combination therapy	
			Post-treatment CZA MIC (mg/L)	Post-treatment AMK MIC (mg/L)	Post-treatment CZA MIC (mg/L)	Post-treatment AMK MIC (mg/L)
CTAP #226	24	8	64	No growth	No growth	No growth
CTAP #226	24	8	96	No growth	No growth	No growth
CTAP #233	24	8	156	No growth	No growth	No growth
CTAP #233	24	8	>256	No growth	No growth	No growth

**Fig 3:**
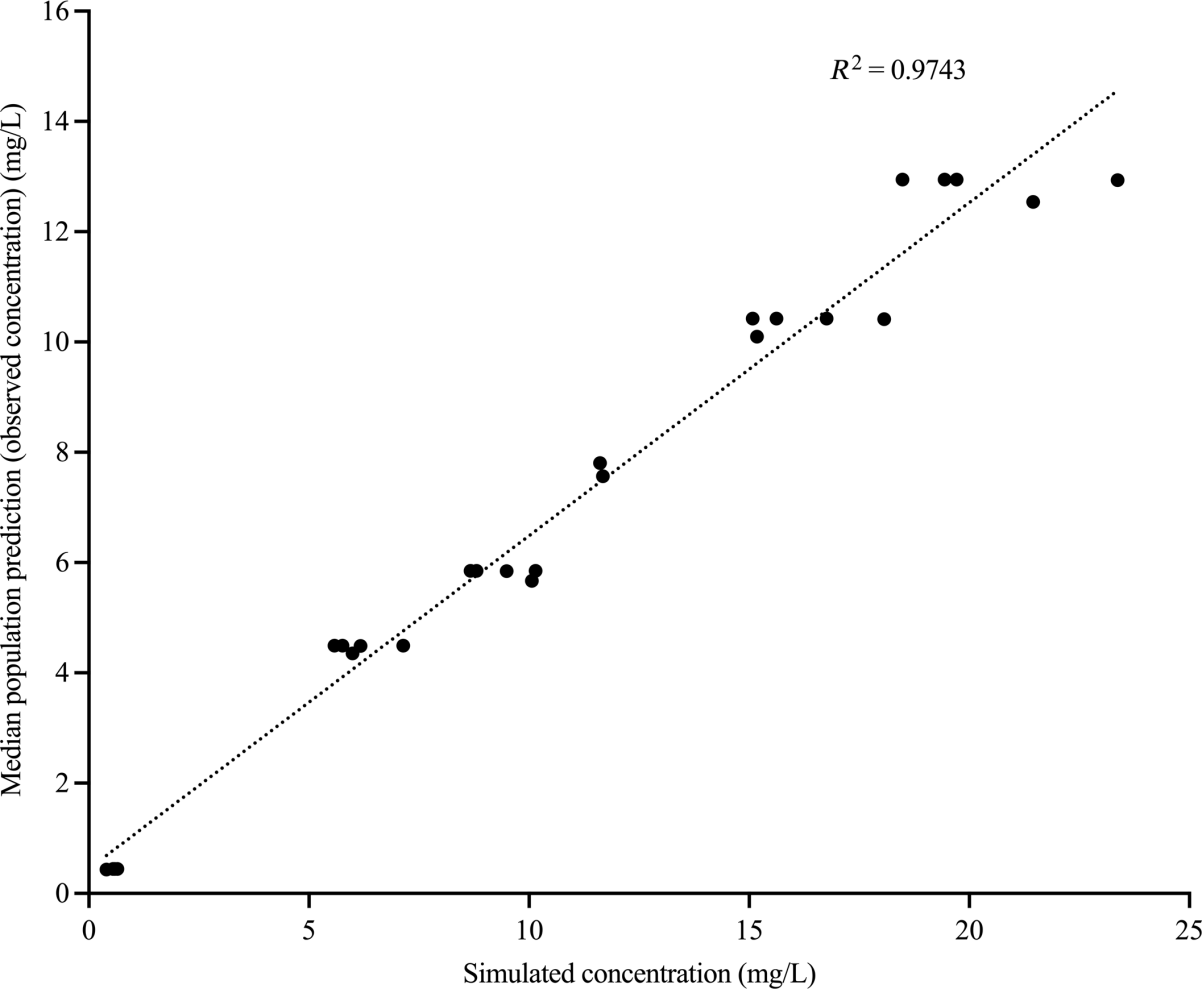
Median population prediction for observed concentrations versus simulated concentration for amikacin.

**Fig 4:**
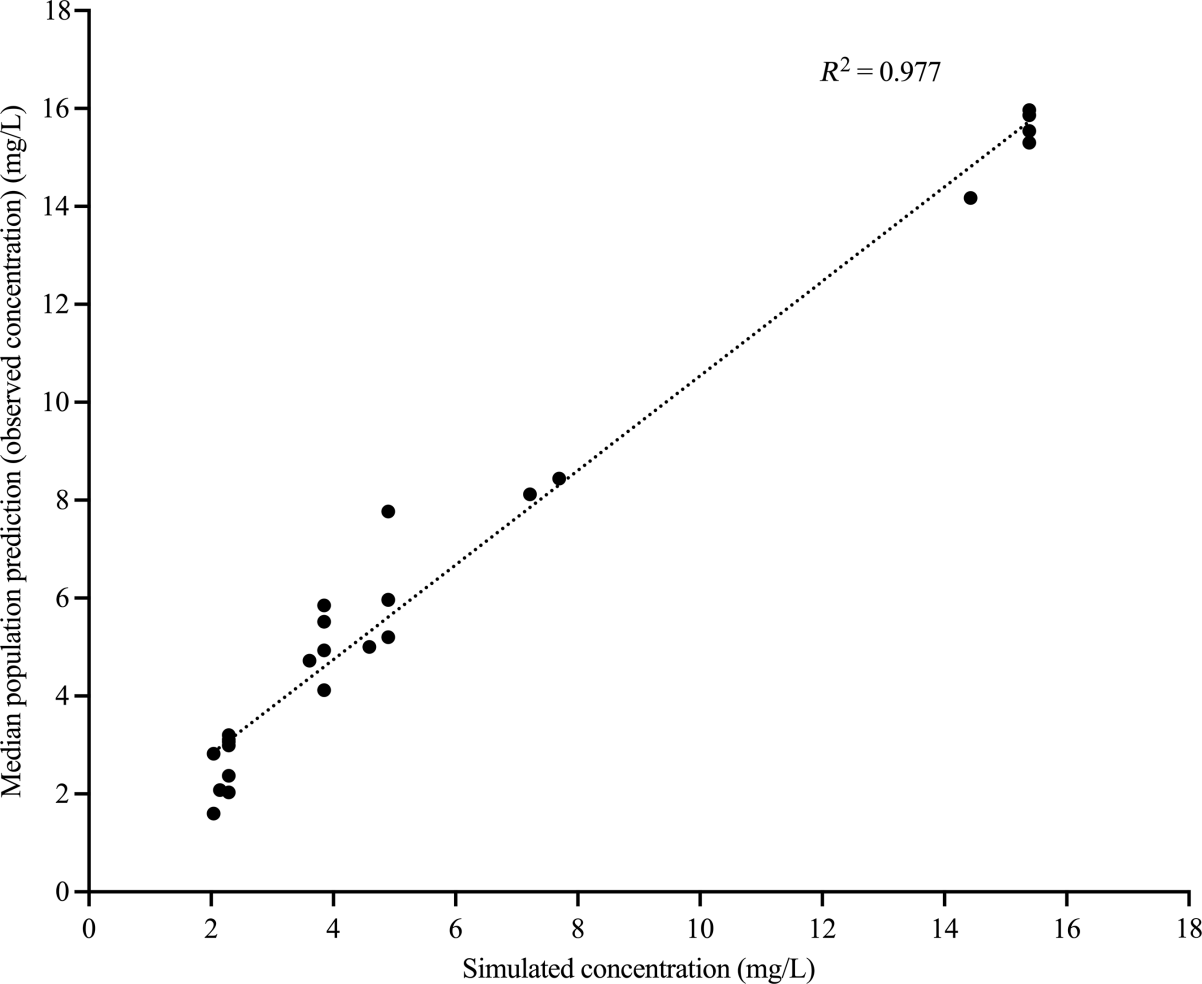
Predicted median population for observed concentrations versus simulated concentration for avibactam.

The relationship between median population prediction for observed concentrations versus simulated concentrations of amikacin and avibactam is presented in Fig. 3 and 4, respectively. Significant degradation in ceftazidime was detected in all monotherapy arms, similar to our previous observation for piperacillin (38).

## DISCUSSION

This study found that a combination of 2 g ceftazidime/0.5 g avibactam with 15 mg/kg amikacin exhibited superior bacterial killing and resistance suppression when compared to monotherapies against two clinical *E. coli* isolates. Though the combination of ceftazidime/avibactam and amikacin has been previously studied on gram-negatives, the study efforts have largely focused on *Pseudomonas aeruginosa* and *Klebsiella pneumoniae*. To the best of our knowledge, this is the first HFIM study of its kind conducted on *E. coli*.

The ceftazidime/avibactam and amikacin combination therapy resulted in bacterial eradication within the first 24 (CTAP #226) 48 h (CTAP #233). This finding is congruent with a study comparing ceftazidime/avibactam and amikacin combination therapy and monotherapy against colistin-resistant gram-negatives, including two ceftazidime/avibactam *E. coli* isolates (17). Using a checkerboard assay, the study found that the combination of ceftazidime/avibactam and amikacin had a susceptible breakpoint index of greater than two, rendering it below the susceptibility breakpoint (17).

Similarly, a HFIM study conducted by Huang et al. (14) investigated the combination of ceftazidime/avibactam and amikacin against *K. pneumoniae* and found that the ceftazidime/avibactam and amikacin combination resulted in superior bacterial killing compared to the amikacin monotherapy; however, they also found that the ceftazidime/avibactam monotherapy achieved comparable bacterial killing compared to the combination therapy. The regrowth observed in amikacin monotherapy by Huang is consistent with our findings, where both isolates exhibited a 3-log reduction within the first 6 h followed by regrowth for the remainder of the experiment. In contrast, ceftazidime/avibactam monotherapy in our study produced a 4-log reduction within the first 6 h, but regrowth was observed as early as 8 h and continued to increase throughout the experiment. It should be noted, in the Huang et al. (14) study, the *K. pneumoniae* isolates had initial ceftazidime/avibactam MICs below the clinical breakpoint and were considered susceptible to ceftazidime/avibactam.

Emergence of resistant subpopulations was most consistently observed in ceftazidime/avibactam arms. This was expected, as prior to the HFIM, both isolates were resistant to ceftazidime/avibactam, possessing an MIC two times the clinical breakpoint. Following the HFIM, the MIC of ceftazidime/avibactam for both isolates was higher than prior to the HFIM. Bioinformatic analyses identified the presence of the metallo-β-lactamase IMP-4, an enzyme capable of hydrolyzing ceftazidime (39). This enzymatic activity correlated with degradation patterns observed in the pharmacokinetic profiles from ceftazidime/avibactam monotherapy arms within the hollow fiber infection model. Introduction of amikacin in combination regimens significantly reduced the extent of ceftazidime degradation, suggesting that the rapid rate of synergistic bacterial killing may have reduced the extent of IMP-4 mediated hydrolysis (Fig. S1 and S2).

Both isolates possessed aminoglycoside acetyltransferase enzymes aac(6′)-Ib4 and aac(3)-lld prior to treatment; despite this, the baseline MIC was within the “susceptible” ECUAST clinical breakpoint parameters, although at the very upper limit. The aac(6′)-Ib4 gene, an acetyltransferase, is considered to be responsible for most amikacin resistance (40, 41). However, Galimand et al. (42) reported that while aac(3)-lld was effective against tobramycin, dibekacin, kanamycin B, sisomicin, gentamicin C1A, and fortimicin, antibiotics including amikacin, kanamycin A, netilmicin, neomycin B, and apramycin were not substrates for the acetyltransferase enzyme.

We acknowledge that there are limitations to this study. While the HFIM is a highly controlled system optimized to represent clinical dosing regimens, it is unable to fully mimic the human environment. Many host factors can impact the pharmacokinetic and pharmacodynamic targets, such as lowering targets due to immune killing or raising them if host factors reduce bacteria susceptibility (43). It is also worth noting that despite many *in vitro* combination therapy successes, reports on the clinical efficacy of combination therapies are still varied (11). Thus, a clinical study investigating the efficacy of a ceftazidime/avibactam-amikacin combination therapy would be necessary to evaluate the combination’s capacity as a carbapenem-sparing agent for drug-resistant *E. coli* infections.

Clinical use of aminoglycosides is constrained by nephrotoxicity and ototoxicity risks, especially with prolonged exposure (44). Of note, repeated dosing in the monotherapy arm did not result in suppression of regrowth, despite substantial initial killing, suggesting that a short course (1 to 3 days) of amikacin may be adequate, particularly when used in a synergistic combination with ceftazidime-avibactam. Such a short course of aminoglycosides is likely to significantly reduce the risk of toxicity, especially when coupled with therapeutic drug monitoring to individualize dosing, maximize efficacy, and minimize toxicity.

### Conclusion

To conclude, this study demonstrated the bacterial killing capacity of ceftazidime/avibactam when used in combination with amikacin against two ceftazidime/avibactam-resistant strains. We found that the drug combination was superior to the monotherapies at bacterial killing, reducing the bacteria to <LOQ (10^2^ CFU/mL), and also inhibiting the emergence of resistant populations. Thus, the combination of ceftazidime/avibactam and amikacin provides a potential alternative to carbapenem antibiotics, a necessary consideration in the age of rapidly developing carbapenem resistance.

## Data Availability

Whole genome sequencing read data have been deposited in the NCBI Sequence Read Archive under BioProject number PRJNA1414808 and accession numbers SAMN54914261, SAMN54914262, SAMN54914263, SAMN54914264, SAMN54914265, SAMN54914266, SAMN54914267, SAMN54914268, and SAMN54914269.

## SUPPLEMENTAL MATERIAL

Supplemental material aac.01377-25-s0001.docx Fig. S1 and S2; Table S1.

ASM does not own the copyrights to Supplemental Material that may be linked to, or accessed through, an article. The authors have granted ASM a non-exclusive, world-wide license to publish the Supplemental Material files. Please contact the corresponding author directly for reuse.
