# Supplementary material for: Evaluating the efficacy of ceftazidime/avibactam plus amikacin combination therapy as a carbapenem-sparing agent against ceftazidime/avibactam-resistant Escherichia coli using a hollow fiber infection model: a proof-of-concept study
Source: Antimicrob Agents Chemother. 2026 May 6;70(6):e01377-25. doi: 10.1128/aac.01377-25 (PMC13231872; doi:10.1128/aac.01377-25)
Supplement: Supplemental material — Fig. S1 and S2; Table S1. [file aac.01377-25-s0001.docx]

Supplemental information

Ceftazidime Degradation

We were able to quantify ceftazidime with chromatographic method presented in the manuscript. However, due to significant enzymatic degradation, particularly in the monotherapy arm, there is poor correlation between simulated profile and observed concentrations after enzymatic degradation. We have previously described this phenomenon in hollow fibre infection model with semi-quantitative analysis of degradation products for piperacillin-tazobactam (Antimicrobial Agents and Chemotherapy 2022, 66:e0016222). Figures S1 and S2 below show the observed ceftazidime concentrations superimposed on the simulated concentration time profile when ceftazidime is administered as a monotherapy or in combination with amikacin, respectively.

Figure S1 Ceftazidime observed versus simulated concentrations when administered alone

Figure S2 Ceftazidime observed and simulated concentration when combined with amikacin

Table S1: Variant calling mapping statistics

| Sample ID | Mapped Read pairs | Mean Coverage |
| --- | --- | --- |
| SF7560 | 6301525 | 366 |
| SF7561 | 4506876 | 247 |
| SF7562 | 4429165 | 245 |
| SF7563 | 2360051 | 293 |
| SF7564 | 5067501 | 272 |
| SF7565 | 4986937 | 275 |
| SF7566 | 5581705 | 306 |
| SF7567 | 5640232 | 324 |
| SF7568 | 6124871 | 352 |
| SF7569 | 5876288 | 339 |
| SF7570 | 6760767 | 388 |
| SF7571 | 4603848 | 253 |
| SF7573 | 4300778 | 234 |
| SF7574 | 3251124 | 180 |
| SF7575 | 158322 | 9 |
